# Infusion Rate Escalation Study of Rituximab in Patients with CD20+ B-Cell Lymphomas: A Single Institution Analysis in Japan

**DOI:** 10.1155/2013/863909

**Published:** 2013-04-03

**Authors:** Masahiro Yokoyama, Yasuhito Terui, Kengo Takeuchi, Eriko Nara, Kenji Nakano, Kyoko Ueda, Noriko Nishimura, Yuko Mishima, Sakura Sakajiri, Naoko Tsuyama, Keiya Ozawa, Kiyohiko Hatake

**Affiliations:** ^1^Department of Hematology, Cancer Institute Hospital, 3-8-31 Ariake, Koto-ku, Tokyo 135-8550, Japan; ^2^Division of Pathology, The Cancer Institute, 3-8-31 Ariake, Koto-ku, Tokyo 135-8550, Japan; ^3^Pathology Project for Molecular Targets, The Cancer Institute, 3-8-31 Ariake, Koto-ku, Tokyo 135-8550, Japan; ^4^Division of Hematology, Department of Medicine, Jichi Medical University, 3311-1 Yakushiji, Shimotsuke, Tochigi 329-0498, Japan

## Abstract

*Background*. To determine the maximum tolerable infusion rate of rituximab, and investigate the safety and feasibility of rapid infusion of rituximab for patients with CD20 positive B-cell lymphomas (CD20+NHL). *Patients and Methods*. 18 patients with CD20+NHL were registered. This study had six cohorts of administration rate of rituximab. The median age was 56 years (range, 38–79), and five of 18 patients were male. Two patients (11%) with diffuse large B-cell lymphoma were receiving R-CHOP therapy, two (11%) with indolent lymphoma were receiving R-CVP therapy, and 14 (78%) with indolent lymphoma were receiving rituximab as maintenance therapy. *Results*. A total of 88 cycles of rituximab was administered. Rapid infusion of rituximab was well tolerated, with only one grade 3 leukocytepenia and one grade 4 neutropenia. Four patients (22%) developed grade 1 infusion-related toxicities at the first administration of rituximab. No patient with severe drug-related events was observed. *Conclusions*. We determined that the maximum tolerable infusion rate of rituximab is 300 mL/h (under 700 mg/h), and confirmed that administration of over 60 minutes is safe and feasible. We recommend rapid administration of rituximab for practice setting in patients with CD20+NHL being treated with rituximab or rituximab-containing chemotherapy. (Clinical trial no. JFCR2009-1027).

## 1. Introduction

Rituximab, a chimeric murine/human IgG kappa monoclonal antibody, has been developed against CD20+ B-cell lymphomas (CD20+NHL) [1]. Adding rituximab to chemotherapy is standard therapy for patients with CD20+ aggressive and indolent B-cell lymphomas (CD20+NHL) and, more recently, as maintenance therapy after response to induction therapy for indolent B-cell lymphoma [2–5]. 

Despite the improved outcome with the addition of rituximab to chemotherapy, its administration can be associated with infusion-related toxicities such as fever, rash, urticaria, dyspnea, bronchospasms, hypertension, hypotension, or other allergic reactions/hypersensitivities. Infusion-related reactions can occur in approximately one-fourth of patients receiving the first administration of rituximab. Recommendations to reduce the incidence of these reactions include the administration of rituximab over a prolonged period, usually 5-6 hours for the first infusion and 3-4 hours for subsequent infusions.

Recently, several investigators have reported that rapid infusion of rituximab, for approximately 60–90 minutes, is a safe administration schedule for the patients with CD20+NHL [6–11]. However, it is unclear what would constitute the fastest, safest, and most convenient administration schedule for rituximab. In this prospective infusion rate escalation study, we determined that the maximum tolerable infusion rate of rituximab produced a simpler administration schedule, and we investigated the safety and feasibility of rapid infusion for patients with CD20+NHL.

## 2. Patients and Methods

### 2.1. Study Design

This study had six cohorts of infusion rate of rituximab and was performed in a single institution, the Cancer Institute Hospital in Japan. Patients received rituximab monotherapy (including maintenance therapy) or rituximab combined with chemotherapy, and they were followed for toxicities until the end of treatment. We mixed rituximab 375 mg/m^2^ and 250 mL of 0.9% normal saline for each patient, which was more than 1 mg/mL of rituximab. At the first cycle of rituximab infusion of all cohorts, rituximab was administered at 10 mL/h for the first 1 hour. The next infusion rate was 50 mL/h for the second hour, and the final infusion rate was 100 mL/h until the end of treatment. After the first infusion, each infusion rate of cohorts was given from the second to a maximum of eight cycles of rituximab infusion (Table 1).

All cohorts of at least three patients were assigned to receive rituximab. If no severe adverse event occurred during the infusion in three patients, the next cohort was opened. In case of a severe adverse event, three additional patients were to be included in the same cohort. If two patients developed severe adverse events, a safety evaluation committee (SEC) was convened, and its members looked for a causal relationship between these events and rapid infusion of rituximab. If the SEC concluded that the infusion rate of this cohort had caused the severe adverse events, the previous infusion rate would be considered the maximum tolerable rate.

The primary endpoint of this study was to assess the maximum tolerable infusion rate of rituximab. The secondary endpoints were to assess feasibility and safety, defined as incidence of toxicities.

### 2.2. Patients

Patients were eligible for enrollment if they were diagnosed with CD20+NHL according to the WHO classification [12] and were at least 20 years old. Patients had to have no other malignancies and to have adequate organ functions. Patients were excluded if they had any one of the following criteria: central nervous system involvement of lymphoma, seropositive for human T-cell lymphoma virus type I antibody or human immunodeficiency virus antibody, pregnancy, Burkitt lymphoma, or other highly aggressive lymphomas. This study was conducted in accordance with the ethical guidelines mandated by the Declaration of Helsinki. All patients signed informed consent that was approved by institutional review board at the Cancer Institute Hospital of Japanese Foundation for Cancer Research (JFCR). (Clinical trial no. JFCR2009-1027.)

All patients received rituximab or rituximab combined with chemotherapy. Maintenance therapy of rituximab was administered at the standard dose of 375 mg/m^2^ in four cycles of weekly infusion [5]. All patients received chlorpheniramine and eveprophene premedication without steroids. If infusion-related toxicities were present, hydrocortisone was used. Antibiotics, antifungals, antivirals, and standard antiemetics were used at the discretion of the treating physicians. If febrile neutropenia was present, levofloxacin or cefepime was used as preemptive therapy. As prophylaxis for *Pneumocystis jiroveci* infection, trimethoprim-sulfamethoxazole was used. The antihepatitis B viral agent entecavir was used as prophylaxis for hepatitis B virus reactivation if patients were seropositive for hepatitis B virus surface antigen.

Biopsy samples from eligible patients were collected at the time of diagnosis. All histopathological samples were reviewed according to the WHO classification [12] by expert hematopathologists (N. Tsuyama and K. Takeuchi).

### 2.3. Toxicity Assessment

Toxicities were evaluated using the National Cancer Institute Common Toxicity Criteria (version 4.0) standard toxicity grading. Clinical examination was done before each cycle, every 30 minutes during infusion, and immediately following each treatment. Electrocardiography (ECG) monitoring was done during every infusion.

## 3. Results

### 3.1. Patients

Between February 2010 and October 2010, 18 patients with diagnosed CD20+NHL were treated at the Cancer Institute Hospital. Baseline clinical characteristics are outlined in Table 2. The median age was 56 years (range 38–79), five of the 18 patients were male, and ECOG performance status of 16 patients was 0. Median administered rituximab was 560 mg (range 498–680). All patients had adequate cardiac function evaluated with the level of brain natriuretic peptide (BNP) and left ventricular ejection fraction (LVEF) by ultracardiography. Median levels of BNP and % of LVEF were 15.1 pg/mL (range 5.8–55.5) and 73% (range 58.3–81.8). Two patients (11%) with diffuse large B-cell lymphoma were receiving R-CHOP therapy. Two patients (11%) had indolent lymphoma, one with follicular lymphoma who was receiving R-CVP therapy and the other with extranodal marginal zone lymphoma who was receiving R-CVP therapy. Fourteen (78%) had indolent lymphoma and were receiving four cycles of rituximab as maintenance therapy.

### 3.2. Safety

All 18 patients were receiving rapid infusion of rituximab and were evaluable for safety analysis. A total of 88 cycles (range 4–8 for each patient) of rituximab was administered. The treatment of rapid infusion of rituximab for the patients with CD20+NHLs was well tolerated. No patients were taken off rapid infusion of rituximab due to toxicities. All toxicities caused by this infusion rate escalation study are listed in Table 3.

#### 3.2.1. Hematological Toxicities

Three patients (17%) developed leukocytopenia, one (6%) with grade 3, and three patients (17%) developed neutropenia, one (6%) with grade 4. Two patients (11%) developed grade 1 anemia, and no patients developed grade 2 to 4 anemia or any grade of thrombocytopenia.

#### 3.2.2. Nonhematological Toxicities

Four patients (22%) developed grade 1 infusion-related toxicities at the first administration of rituximab. All of those patients had recovered from their infusion-related adverse events, and they continued in this study.

No patients developed grade 2 to 4 antibody infusion-related toxicities, and no grade 3 to 4 nonhematological adverse events were associated with this study.

### 3.3. Maximum Tolerable Infusion Rate and Recommended Infusion Rate of Rituximab

All of the 6 cohorts did well, without severe adverse events. All adverse events were confirmed at the SEC. In cohort 6, each body surface area of three patients was 1.82 m^2^, 1.70 m^2^, and 1.39 m^2^, and they received each infusion volume of rituximab, 642 mg/h, 604 mg/h, and 517 mg/h, instead of the same infusion rate of 300 mL/h.

The maximum tolerable infusion rate of rituximab was not clarified, but for the final cohort, 300 mL/h (under 700 mg/h) of administration rate was safe and feasible. We determined that the recommended infusion rate of rituximab is 300 mL/h (under 700 mg/h) over 60 minutes in this study, if patients receive the administered first cycle without severe infusion-related toxicities.

## 4. Discussion

 For patients with CD20+NHL, treatment with rituximab has improved their outcomes, but there is still room for improvement. In the present paper, we focused on convenience for the patients in the ambulatory therapy center as well as on improved utilization of health care resources. Faster administration of rituximab could reduce the comedical staff workload and increase convenience for patients in the center. We wanted to find out whether we could administer rituximab faster and still maintain safety, not increasing toxicities. 

To reduce rituximab infusion times, we aimed to determine the maximum tolerable infusion rate of rituximab. The absolute maximum rate of rituximab was not determined, but the maximum infusion rate of cohort 6 (over 60 minutes) for patients with CD20+NHL was well tolerated and feasible. Therefore, we determined that the maximum tolerable infusion rate of rituximab is 300 mL/h (under 700 mg/h) in this sduty.

Sehn et al. reported a prospective study of 150 patients receiving rituximab with corticosteroid-containing chemotherapy and 56 patients receiving rituximab as maintenance therapy in 90-minute. More than 1,200 patients have been treated with 90 minutes infusion of rituximab in the community setting of British Columbia Cancer Agency and University of British Columbia [7].

Salar et al. also reported 70 patients receiving rapid infusion of rituximab over 90 minutes with or without steroid premedication [6]. Tuthill et al. described 54 patient administered rituximab over 60 minutes of constant infusion with hydrocortisone [9]. Chiang first showed a prospective study in Asia with over 90 minutes of infusion in 79 patients with a total of 269 administrations of rituximab [11]. Siano et al. determined the maximum tolerated infusion rate of rituximab as being 700 mg/h over 60 minutes without steroid premedication [8]. In these previously published studies, rapid infusion of rituximab, approximately 60–90 minutes, was a safe administration schedule for patients with CD20+NHL. Our present study reconfirmed that rapid infusion of rituximab (300 mL/h, under 700 mg/h) over 60 minutes of constant infusion is well tolerated by patients with CD20+NHL.

As for safety management, we found the incidence of leukocytopenia and neutropenia to be three (17%) and three (17%). We decided that leukocytopenia and neutropenia were not related to the rapid infusion of rituximab, but rather to combined chemotherapies. In addition, four patients (22%) who had grade 1 infusion-related toxicities were observed at the first administration of rituximab. These allergic reactions might have been related to rituximab, not related to rapid infusion because of unaltered administration at the first cycle. No patients with severe adverse events were observed in this study.

Siano et al. reported that BNP values increased 24 hours after rapid infusion of rituximab. Although the levels of BNP returned baseline values, this elevated BNP could be related to the volume overload caused by the fast infusion. On the other hand, Provencio et al. reported that LVEF of patients with non-Hodgkin lymphoma decreased after treatment with rapid infusion of rituximab [10]. Although we did not evaluate the level of BNP and percent of LVEF for eligible patients after treatment with rapid infusion of rituximab, we observed no patients with any symptoms of cardiac decompensation or with severe arrhythmias as monitored by ECG during administration. However, in elderly patients or patients who have decreased cardiac function, we should monitor rapid administration of rituximab for potential volume overload or cardiogenic symptoms.

In conclusion, we determined that the maximum tolerable infusion rate of rituximab is 300 mL/h (under 700 mg/h) and confirmed that administration of over 60 minutes is safe and feasible. Shortening of rituximab administration times has reduced comedical staff workload and is more convenient for patients in the ambulatory therapy center. We recommend rapid administration of rituximab over 60 minutes in practice settings for patients with CD20+NHL receiving rituximab or rituximab-containing chemotherapy.

## Figures and Tables

**Table 1:** Administration schedule for each cohort of rapid infusion rituximab.

Rituximab 375 mg/m^2^ intravenously in 250 mL normal saline	
Cycle 1	
10 mL/h (first 1 hour) → 50 mL/h (second 1 hour) → 100 mL/h (until end of treatment)	
Cycle 2–8	
Cohort 1	50 mL/h (constant infusion, until end of treatment)
Cohort 2	100 mL/h (constant infusion, until end of treatment)
Cohort 3	150 mL/h (constant infusion, until end of treatment)
Cohort 4	200 mL/h (constant infusion, until end of treatment)
Cohort 5	250 mL/h (constant infusion, until end of treatment)
Cohort 6	300 mL/h (constant infusion, until end of treatment)

**Table 2:** Patient characteristics.

Characteristics	Number of cases	%
Patients	18	
Median years of age (range)	56 (38–79)	
Gender, male/female	5/13	28/72
ECOG PS, 0/1	16/2	89/11
Median body height of patients (range)	157.5 cm (147.3–175)	
Median body weight of patients (range)	50.6 kg (39.9–76.5)	
Median body surface area of patients (range)	1.5 m^2^ (1.33–1.83)	
Median quantity of administered R (range)	560 mg (498–680)	
Cardiac function (pretreatment)		
Median level of BNP (range)	15.1 pg/mL (5.8–55.5)	
Median % of LVEF (range)	73% (58.3–81.8)	
Diagnosis		
Follicular lymphoma (FL), grade 1	10	56
FL + diffuse large B-cell lymphoma	2	11
Extranodal marginal zone lymphoma	1	6
Nodal marginal zone lymphoma	1	6
Waldenström's macroglobulinemia	1	6
Mantle cell lymphoma	1	6
Diffuse large B-cell lymphoma	2	11
Regimens		
R-CHOP	2	11
R-CVP	2	11
R (as maintenance therapy)	14	78
Total cycles of R (range, for each patient)	88 (4–8)	

ECOG: Eastern Cooperative Oncology Group; PS: performance status; R: rituximab; BNP: brain natriuretic peptide; LVEF: left ventricular ejection fraction; R-CHOP: rituximab, cyclophosphamide, doxorubicin, vincristine, and predonisolone; R-CVP: rituximab, cyclophosphamide, vincristine, and predonisolone.

**Table 3:** Adverse events.

	G1		G2		G3		G4		Total	
	*n*	%	*n*	%	*n*	%	*n*	%	*n*	%
Leukocytopenia	1	6	1	6	1	6	0	0	3	17
Neutropenia	1	6	1	6	0	0	1	6	3	17
Anemia	2	11	0	0	0	0	0	0	2	11
Infusion reaction	4	22	0	0	0	0	0	0	4	22
Conduction disorder	1	6	0	0	0	0	0	0	1	6
Ventricular arrhythmia	2	11	0	0	0	0	0	0	2	11
Malaise	3	17	0	0	0	0	0	0	3	17
Hypotension	1	6	0	0	0	0	0	0	1	6
Pharyngitis	2	11	1	6	0	0	0	0	3	17
Bronchitis	0	0	1	6	0	0	0	0	1	6
Herpes zoster infection	0	0	1	6	0	0	0	0	1	6
Diarrhea	1	6	1	6	0	0	0	0	2	11
AST increased	3	17	0	0	0	0	0	0	3	17
ALT increased	2	11	0	0	0	0	0	0	2	11
Nausea	1	6	0	0	0	0	0	0	1	6
Palpitations	1	6	0	0	0	0	0	0	1	6
Allergic rhinitis	1	6	0	0	0	0	0	0	1	6
Constipation	1	6	1	6	0	0	0	0	2	11
Insomnia	2	11	0	0	0	0	0	0	2	11
Peripheral neuropathy	2	11	0	0	0	0	0	0	2	11
Creatinine increased	1	6	0	0	0	0	0	0	1	6
Acne	0	0	1	6	0	0	0	0	1	6
Anorexia	1	6	0	0	0	0	0	0	1	6

AST: aspartate aminotransferase; ALT: alanine aminotransferase.
